# How the built environment affects the spatiotemporal pattern of urban vitality: A comparison among different urban functional areas

**DOI:** 10.1007/s43762-022-00069-4

**Published:** 2022-11-03

**Authors:** Shuwei Tang, Na Ta

**Affiliations:** 1 Key Laboratory of Geographic Information Science, Ministry of Education, East China Normal University, Shanghai, 200241 China; 2 School of Geographic Sciences, East China Normal University, Shanghai, 200241 China; 3 Key Laboratory of Spatial-Temporal Big Data Analysis and Application of Natural Resources in Megacities, Ministry of Natural Resources, No. 500 Dongchuan Road, Shanghai, 200241 China

**Keywords:** Urban vitality, Vitality intensity, Vitality variability, Nighttime vitality, Built environment

## Abstract

Urban vitality is an essential indicator of an area’s capacity to promote lively social and economic activities. Urban functional areas can play different roles throughout the day, and urban vitality may exhibit significant intraday temporal dynamics. However, few studies have evaluated the dynamic vitality throughout the day among various urban functional areas or explored how the built environment influences this attribute. To bridge this gap, we assessed the vitality dynamics in intensity, variability, and night ratio. We then examined the influencing factors of urban vitality in Central Shanghai using heatmap and point of interest (POI) data. We found significant differences in the intensity, variability, and night ratio of urban vitality among different urban functional areas. The difference in vitality intensity was more significant than the variability and night ratio between weekdays and weekends. The built environment significantly affected urban vitality, but its role differed among the various urban functional areas. Overall, describing urban vitality from a dynamic perspective could improve our understanding of the differences in attracting and maintaining human activities among different urban functional areas.

## Introduction

Urban vitality refers to the capacity of a place to promote lively social and economic activities (Jacobs, 1961). Increasingly, scholars and managers have aimed to enhance urban vitality through urban planning (Xia et al., 2022; Ye et al., 2018). Vibrant cities tend to attract more high-end talent, promote economic development, enhance the subjective well-being of residents, and increase urban competitiveness (Mouratidis & Poortinga, 2020; Woodworth & Wallace, 2017; Zeng et al., 2018).

The emergence of big data provides new opportunities to analyze the quantitative characteristics of urban vitality (Jin et al., 2017; Sulis et al., 2018). Traditional data such as questionnaire data or case studies can often only analyze static characteristics of vitality at small spatial scales (Maas, 1984; Sung & Lee, 2015; J. Wu et al., 2018a, 2018b), which limits our understanding of the delicate spatial and temporal structures of the urban vitality. In recent years, with the development of information and communication technology (ICT), large-scale, high-precision, and multidimensional data have provided the latest support for the study of urban vitality (Batty, 2010). Over the past decade, based on mobile phone data (Yue et al., 2017), nighttime light data (Zheng et al., 2017), check-in data (Gan et al., 2021; He et al., 2018), etc., scholars have quantitively analyzed the spatial distribution characteristics of urban vitality at the neighborhood, urban block, and kilometer grid scales (C. Wu et al., 2018a, 2018b; Ye et al., 2018; Zhang et al., 2021).

Compared to spatial characteristics, the temporal dynamics of urban vitality have received less attention. Most studies have more notably focused on the variation in urban vitality between spatial units, while little attention has been given to the characteristics of human activities over time in each spatial unit (Liu et al., 2019; Xia et al., 2022). For Jacobs, urban vitality meant that many people walked through neighborhoods at different times for various activity purposes (Jacobs, 1961). The spatiotemporal stability of human activities at a given location has been considered a fundamental trait of vitality (Sulis et al., 2018). Therefore, the intensity of urban vitality is essential, but the variation in vitality should also be considered. Currently, research on the temporal dimension of urban vitality mainly manifests in two aspects. One type entails the measurement of the intensity of urban vitality within different spaces considering the cumulative urban vitality value over time (Yue et al., 2017), which remains a spatial comparison. The other type involves the analysis of the spatiotemporal patterns of urban vitality through the construction of various indicators, such as the spatiotemporal vitality (C. Wu et al., 2018a, 2018b), variability (Guo et al., 2021; Sulis et al., 2018), and daytime-nighttime vitality (Kim, 2020; Wu et al., 2022; Xia et al., 2022). These indicators can suitably capture urban activities that rise and decline in different time frames.

Urban vitality is a social performance indicator closely related to urban space; therefore, many scholars have explored the influence of the urban built environment on vitality. According to Jane Jacobs' understanding of urban vitality, the five main conditions for a city to remain vital include mixed land use conditions, small-scale blocks, mixed elements, aged buildings, and high-density pedestrian volumes (Jacobs, 1961). In urban morphology theory, it has also been suggested that suitable accessibility, appropriate building density and design, and sufficient functional mix are critical indicators for urban vitality enhancement (Montgomery, 1998). In empirical studies, accessibility, density, typology, and diversity have been highlighted as important spatial factors of urban vitality (Chen et al., 2021; Long & Huang, 2019; Wu et al., 2022; Ye et al., 2018; Yue et al., 2017; Zhang et al., 2021). However, most studies have focused on the influencing factors of general urban vitality or time-specific vitality (Guo et al., 2021; Xia et al., 2022), but few studies have investigated the effects of the built environment on temporal variations in urban vitality.

The importance of urban functions on urban vitality has received much attention among spatial factors. Jane Jacobs argued that a good mix of functions in urban areas could ensure the flow and density of people and activities (Jacobs, 1961). High-vitality places are often equipped with multiple functions (Liu et al., 2019; Yue et al., 2019), while different urban functional areas, such as residential, commercial, and public spaces, play varying roles throughout the day (Liu et al., 2012), leading to various patterns of urban vitality. It is necessary to examine the characteristics of urban vitality among different types of functional areas and analyze its influencing factors.

Therefore, choosing Shanghai Central City as a case study, this paper examined the spatiotemporal characteristics of urban vitality and its influencing factors among different types of urban functional areas. This paper intended to contribute to the literature in two ways. The first was to examine the differences in the intensity, hourly variation, and nighttime vitality level in urban vitality among the different urban functional areas. The second was to analyze the influencing factors of urban vitality variation among different urban functional areas.

## Data and methods

### Study area

**Fig. 1:**
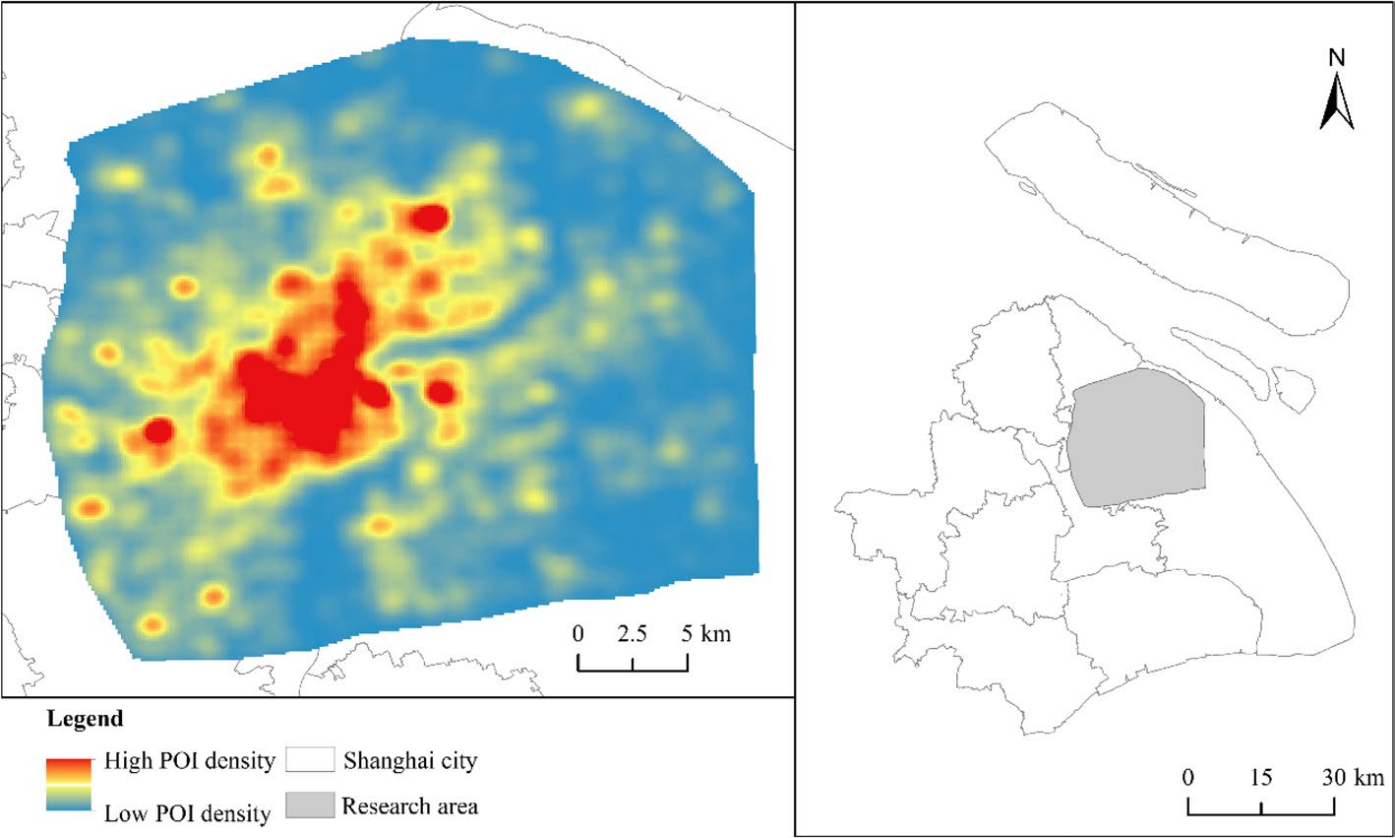
Research area

The main research area in this paper is the central city of Shanghai, as shown in Fig. 1. Shanghai is one of the most advanced cities in China, attracting a large amount of economic activities and population in the last decades. In 2020, Shanghai harnessed a gross domestic product of 3.9 trillion RMB with a population of 27.1 million. In recent decades, the city has attracted many people from all over China seeking working and living opportunities. Urbanization and economic development have significantly increased urban vitality, especially in the central city (Liu et al., 2012; Yue et al., 2019). According to the Shanghai Master Plan (1999–2020), the region within the Outer Ring Road is called the central city, covering a total area of 691.2 square kilometers (Li et al., 2018; Yue et al., 2019) (Fig. 1). Undergoing dramatic spatial reconstruction, the central city of Shanghai has evolved into a livable area with high density and multifunctional land use (Li et al., 2018; Tian et al., 2017). Different land use types show significant spatial and temporal differences in attracting human activities. Thus, the central city of Shanghai could represent a suitable case to investigate the spatial and temporal patterns of urban vitality and its relationship to urban functional areas.

### Urban function measurement based on points of interest (POIs)

Drawing on literature (Hu & Han, 2019; Yuan et al., 2012), this paper measured urban functions based on Amap (http://www.amap.com) POI data. With the help of our technical cooperators, we obtained 1,308,813 POI points of twenty categories in Shanghai using the API interface provided by Amap. Considering the needs of urban vitality research, we deleted records outside central city area. Then, we reclassified the original twenty categories of POIs into five: administration and public service, commercial and business, industrial, residential, and transportation facilities.

**Fig. 2:**
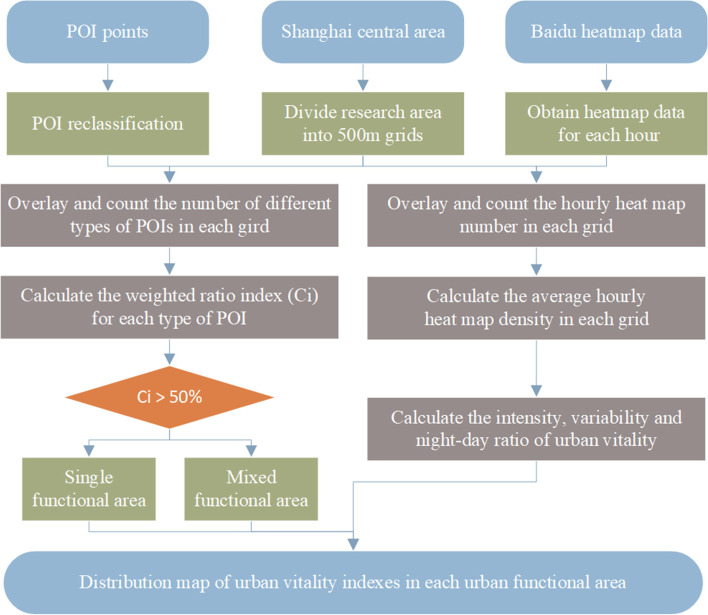
Framework of the process

The urban functional area identification process is shown in Fig. 2. We chose a 500-m grid as the fundamental analysis unit. After reclassification, the five types of POI data were overlaid with 500-m grid data in the central area, and the number of POIs in each grid was counted.

We constructed the POI category ratio index (Ci) to identify the main function in each grid based on the weighted sum of the different types of POIs (Hu & Han, 2019). First, considering the differences in size and importance of the various POI types, the weighted sum (S_i_) of each type of POI was calculated. Then, C_i_ was calculated as the proportion of S_i_ to the total weighted sum of the various POIs, as follows:

1 $$\begin{array}{c}Weighted\;sum\;of\;POI\;type\;i:\;{S}_{i}=\sum {p}_{ix}\end{array}$$

2 $$\begin{array}{c}Category\;ratio\;index\;of\;POI\;type\;i:\; {C}_{i}=\frac{{S}_{i}}{\sum_{i=1}^{5}{S}_{i}}\end{array}$$

where i was the POI category, and p_ix_ denoted the weighted sum of x subtypes of POIs under i categories.

The urban functional area could be determined based on the C_i_ index (Hu & Han, 2019). When the C_i_ value of one POI type in a grid exceeded 50%, this grid was labeled as a single functional area of POI type i. When all C_i_ values were less than 50%, this unit was considered a mixed functional area. The grid was regarded as a no-data area when any POIs of these five categories were not included in a grid unit. Most of the non-data areas were around the Huangpu river.

3 $$\begin{array}{c}Urban\;functional\;area: \;F=\left\{\begin{array}{c}Functional\;area\;i,\;if\;{C}_{i}\ge 0.5\\ Mixed\;functional\;area,\;if\;{ C}_{i}<0.5\end{array}\right.\end{array}$$

**Fig. 3:**
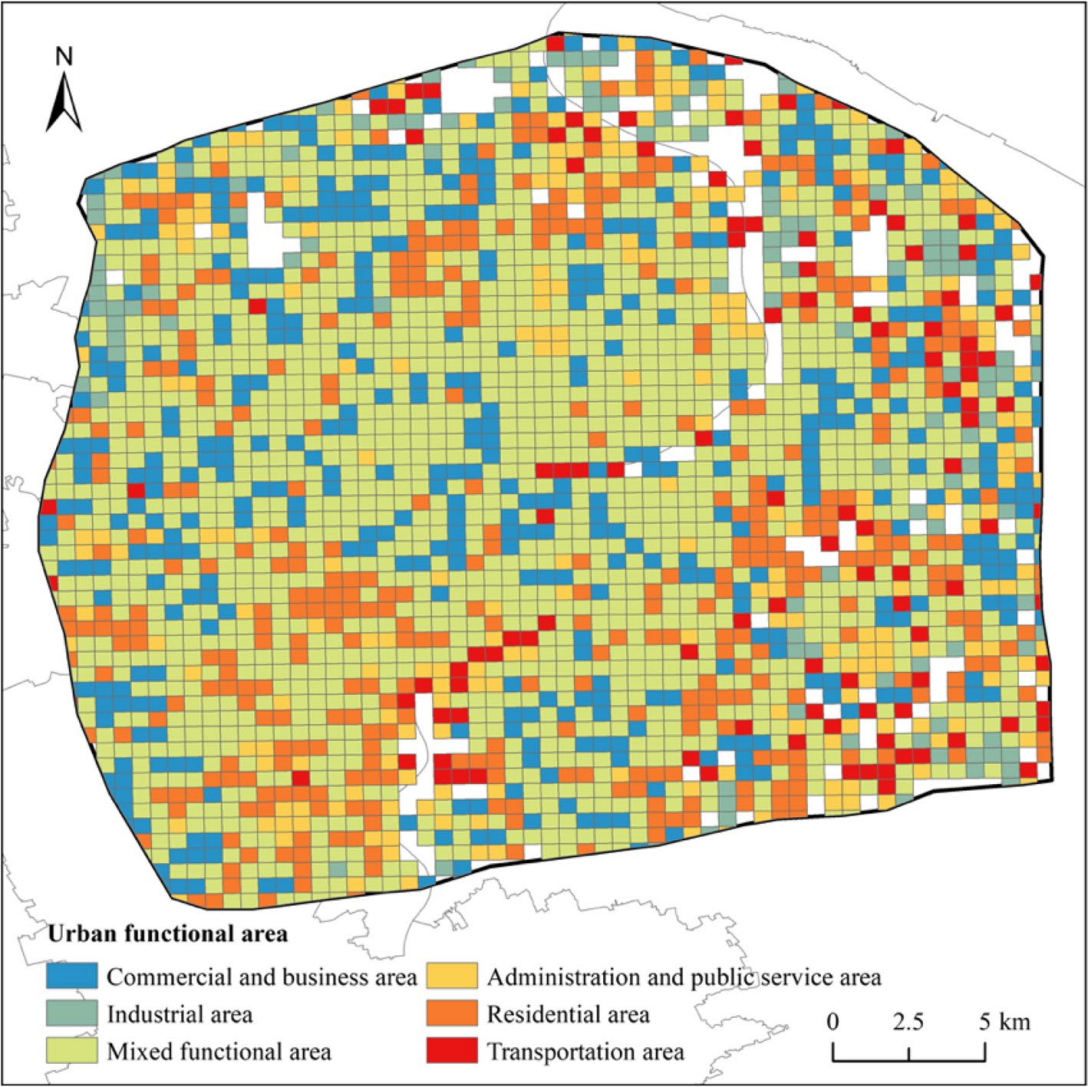
Urban functional areas in Central City of Shanghai

Thus, Central City of Shanghai adopted the mixed functional area as its main functional form, with a proportion of 49.38% (Fig. 3). Regarding single functional areas, commercial and business areas occupied the most significant proportion of 16.80%, followed by residential, administration and public service, industrial, and transportation areas. In addition to typical commercial centers such as Lujiazui, Xujiahui, and People's Square, small commercial areas near the outer ring area could be identified. Residential areas were mainly located in peripheral areas, with a larger proportion of industrial areas distributed in Pudong and a widespread distribution of administration and public service areas.

### Urban vitality measurement based on heatmap data

Baidu heatmaps are based on the geographic location data of cell phone users on this location-based services (LBS) platform. Considering billions of user data, we could suitably obtain the crowd concentration degree during different periods and determine the urban space usage. Hourly heatmap data from 2020.11.21 to 2020.11.27 for Central City of Shanghai were obtained. Though long-term data is better for detecting more complex urban vitality variations, a one-week time frame is usually used to analyze human activities and daily urban vitality variations under a typical urban rhythm cycle (Ettema & van der Lippe, 2009; Guo et al., 2021; Liu et al., 2012, 2019; Raux et al., 2016).

Then, three indexes were calculated to measure urban vitality's spatial and temporal patterns, including the intensity, variability, and night ratio. To construct these indexes, heatmap density in the ith hour (HD_i_) in each grid was calculated as the average heat value per square kilometer for each hour.

First, the intensity was used to measure the mean heatmap density in each grid (Eq. 4).

4 $$\begin{array}{c}Intensity: I=\frac{1}{T}{\sum }_{i=1}^{T}{HD}_{i},\end{array}$$

where T is the total hours in a day and HDi is the heatmap density in the ith hour.

Second, the variability was defined as the difference in heatmap density among the different hours within each grid. This index was used to measure the temporal variation in human dynamics throughout the day: the higher the variability was, the higher the variation over time. The variability could be calculated at the hourly level by comparing the standard deviation of the hourly heatmap density and the intensity of urban vitality (Eq. 5).

5 $$\begin{array}{c}Variability: V=\frac{\sqrt{\frac{1}{T}{\sum }_{i=1}^{T}\;{\left({HD}_{i}-I\right)}^{2}}}{I}\end{array}$$

Third, the night ratio was employed to analyze the performance of the nighttime vitality. A higher night ratio value indicates that more human activities are conducted at night, which reflects the development of nighttime economics and social activities. The night ratio can be calculated as follows:

6 $$\begin{array}{c}Night\;ratio: NR=\frac{{\sum }_{22}^{24}\;{HD}_{i}}{{\sum }_{22}^{24}\;{HD}_{i}+{\sum }_{10}^{12}\;{HD}_{i}}\end{array}$$

All the indicators were calculated for an average weekday and weekend, respectively. These three indexes could jointly reveal the differences in spatial and temporal patterns across the Central City of Shanghai. Areas exhibiting a higher intensity and lower variation could be considered places with a higher degree of vitality, attracting many people throughout the day.

### Built environment measurement

We employed a series of indicators capturing the built environment in each grid. The POI density and diversity reflect the number and mix, respectively, of urban facilities. The average building height was calculated as the height per building in each grid. The road network density, the total road length per square kilometer in each grid, was used to characterize the urban design. The bus stop density reflected the public transit accessibility.

### Model specification

We applied a series of Ordinary Least Squares (OLS) models to evaluate the relationship between the built environment and urban vitality indicators. Due to the skewed distribution of urban vitality index variables, the Box-Cox transformation was employed to convert these variables to approximate normality. The formula for Box-Cox transformation is shown in formula 7(Box & Cox, 1964). The dependent variables were Box-Cox transformed intensity, Box-Cox transformed variability, and Box-Cox transformed night ratio of urban vitality, named as Tran-I, Tran-V, and Tran-NR. The $\uplambda$ values were 0.376, -0.914, and 1.354, respectively.

7 $$\begin{array}{c}Y\left(\uplambda \right)=\left\{\begin{array}{c}\left({Y}^{\uplambda }-1\right)/\uplambda ,\uplambda \ne 0\\ \mathrm{ln}Y,\lambda =0\end{array}\right.\end{array}$$

## Results

### Spatiotemporal distribution of the overall urban vitality

**Fig. 4:**
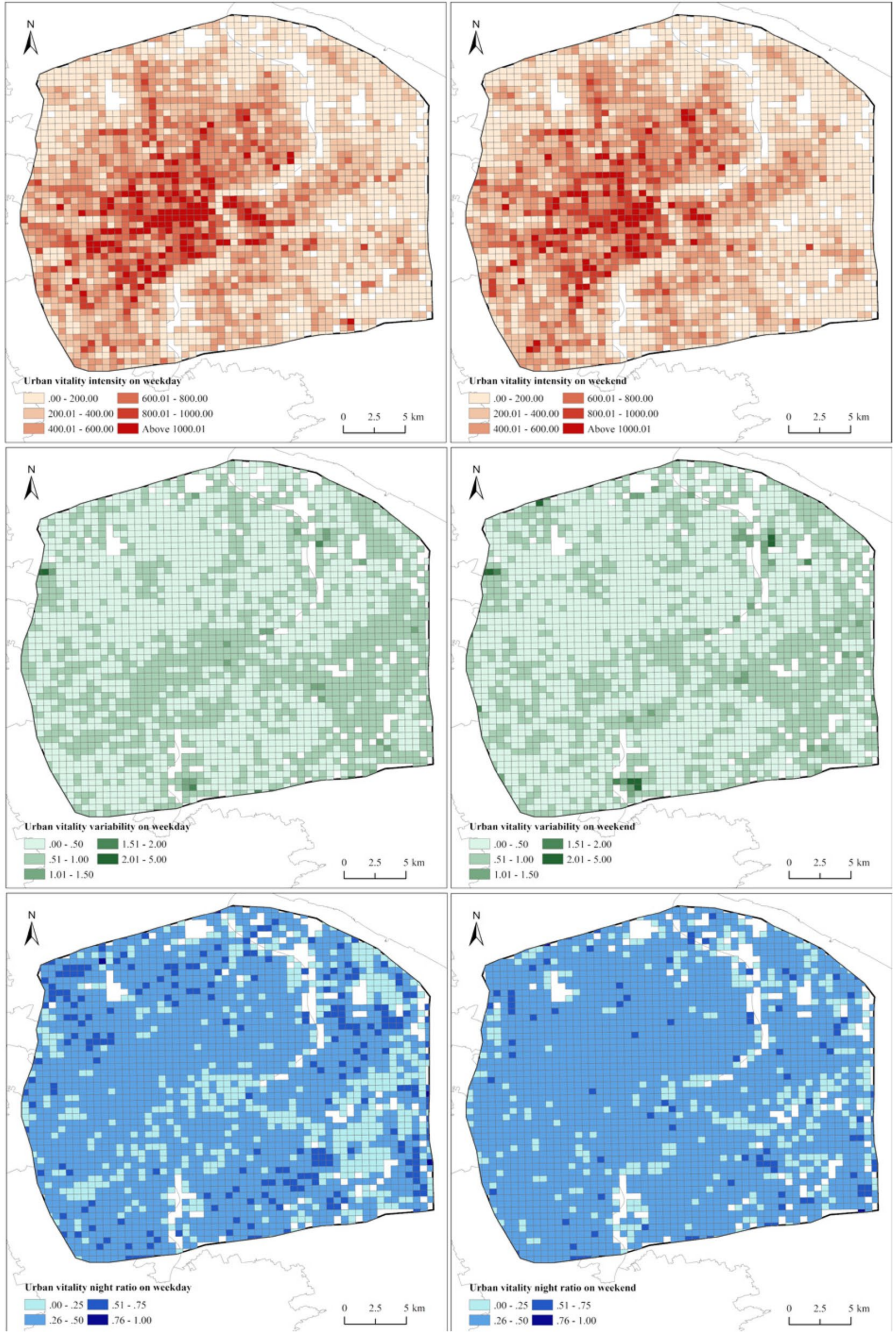
Urban vitality on an average weekday and weekend

Figure 4 shows the status of the urban vitality intensity, variability, and night ratio on an average weekday and weekend. On weekday, the average intensity reached 363.2, the average variability was 0.53, and the average night ratio reached 0.35. The spatial differences in urban vitality were noticeable. The intensity of the urban vitality was higher in the inner-city area centered on People's Square and decreased from the center to the periphery. The intensity in Puxi was higher than that in Pudong. The variability of the urban vitality in most areas varied between 0 and 1, and only a few places exhibited a variability above 1. Spatially, the variability was higher in the inner city and Pudong and lowered in the peripheral regions of Puxi. The night ratio of the urban vitality in most areas varied between 0 and 0.5, indicating that the nighttime vitality was lower than the daytime vitality. Areas with a high daytime vitality mainly occurred in the inner city and Pudong, while areas with a higher nighttime vitality were primarily located in the peripheral residential areas.

The urban vitality on an average weekend was slightly lower than that on the weekday, with an intensity of 341.2. At the same time, the variability and night ratio were comparable to those on the weekday, with mean values of 0.52 and 0.35, respectively (Fig. 4). The spatial distribution of urban vitality during the weekend was similar to that on the weekday. However, there were fewer areas with high vitality, especially in Pudong. The variability decreased in the inner city but increased in peripheral areas during the weekend. Places with the lowest and highest night ratios decreased, while regions with average night ratios increased. This indicates that the diurnal difference in human activities declined during the weekend.

According to the above comparison, it could be found that there existed noticeable spatial and temporal differences in urban vitality. The areas with higher vitality and lower variability values remained the same between an average weekday and weekend, related to the distribution of functional areas in the Central City of Shanghai, where commercial and business areas and entertainment functions are intertwined. Grids with lower vitality and higher variability values were mainly in peripheral regions.

### Urban vitality in the different urban functional areas

Significant differences existed in the intensity, variability, and night ratio of the urban vitality among the various urban functional areas (Table 1). This finding reflects the differences in attracting human activities among the different urban functional areas. Commercial and business areas and mixed functional areas attained higher intensity levels, indicating their higher ability to attract human activities. At the same time, their urban vitality did not vary much throughout the day, and a certain level of human activities was maintained even at night. Residential areas exhibited the highest stability throughout the day and a higher level of vitality at night, which is consistent with the rhythm of urban life. Although the vitality in administration and public service areas and transportation areas reached the medium level, these areas exhibited higher variability and lower vitality values at night, which may be closely related to the service hours of these facilities. Industrial areas attained the lowest vitality values and exhibited more significant variability due to their production characteristics.

**Table 1:** Urban vitality indexes among the various urban functional areas

Urban functional area	Intensity		Variability		Night ratio	
	Weekday	Weekend	Weekday	Weekend	Weekday	Weekend
**Administration and public service area**	231.22	188.13 **	0.62	0.62	0.31	0.31
**Commercial and business area**	455.23	443.50	0.55	0.56	0.34	0.34
**Residential area**	295.79	298.13	0.48	0.48	0.40	0.38 ***
**Industrial area**	85.99	69.84 *	0.67	0.71	0.29	0.31
**Transportation area**	146.52	130.22	0.62	0.62	0.31	0.31
**Mixed functional area**	423.43	393.25 **	0.50	0.48***	0.36	0.37

The statistical significance between an average weekday and weekend is indicated as follows: *** *p* < 0.01, ** *p* < 0.05, and * *p* < 0.1

**Fig. 5:**
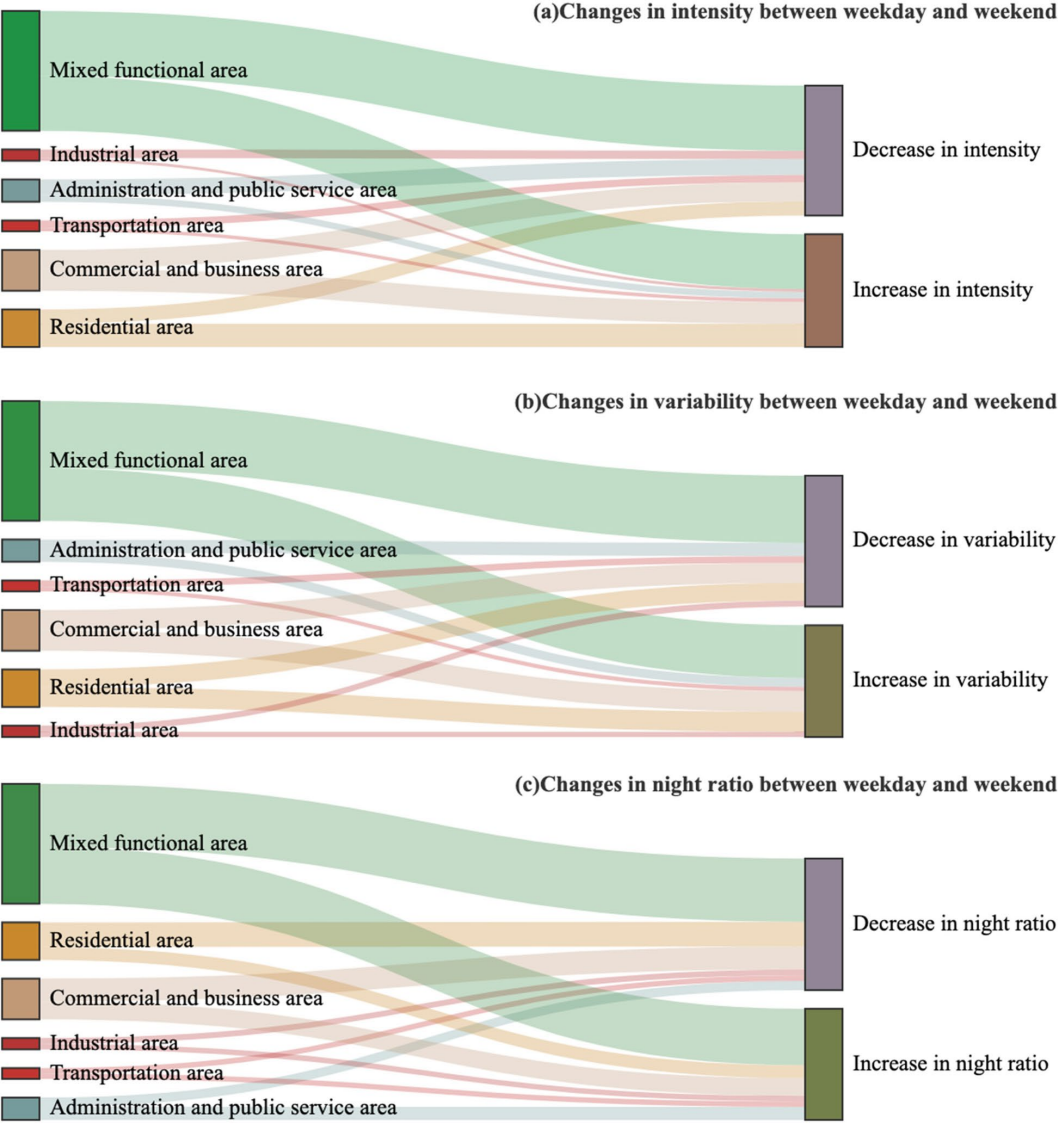
Changes in the urban vitality between an average weekday and weekend

Table 1 and Fig. 5 show the spatial pattern of urban vitality between an average weekday and weekend. Regarding the intensity, in administration and public service areas, industrial areas, and mixed functional areas, the urban vitality intensity values on the weekday were statistically significantly higher than those during the weekend, while the intensity did not significantly differ among the other functional areas. Figure 5a also shows that more grids in these three functional areas exhibited a decreasing trend in the intensity value from weekday to weekend. In comparison, slightly more grids in business and commercial areas and residential areas showed an increasing trend. In terms of the variability, no statistically significant differences existed in the variability of the urban vitality between the weekday and weekend except for mixed functional areas. However, as shown in Fig. 5b, slightly more grids in administration and public service areas, industrial areas, mixed functional areas, and transportation areas exhibited a higher variability on the weekday than during the weekend. For the night ratio, the values in residential areas significantly differed between the weekday and weekend, indicating that the difference between the daytime and nighttime decreased during rest days. As shown in Fig. 5c, more grids in commercial and business areas, mixed functional areas, residential areas, and transportation areas exhibited higher night ratios on the weekday than during the weekend. This suggests a day-to-day difference in the urban vitality in the various urban functional areas. This variation may be influenced by the built environment characteristics in the different functional areas.

**Fig. 6:**
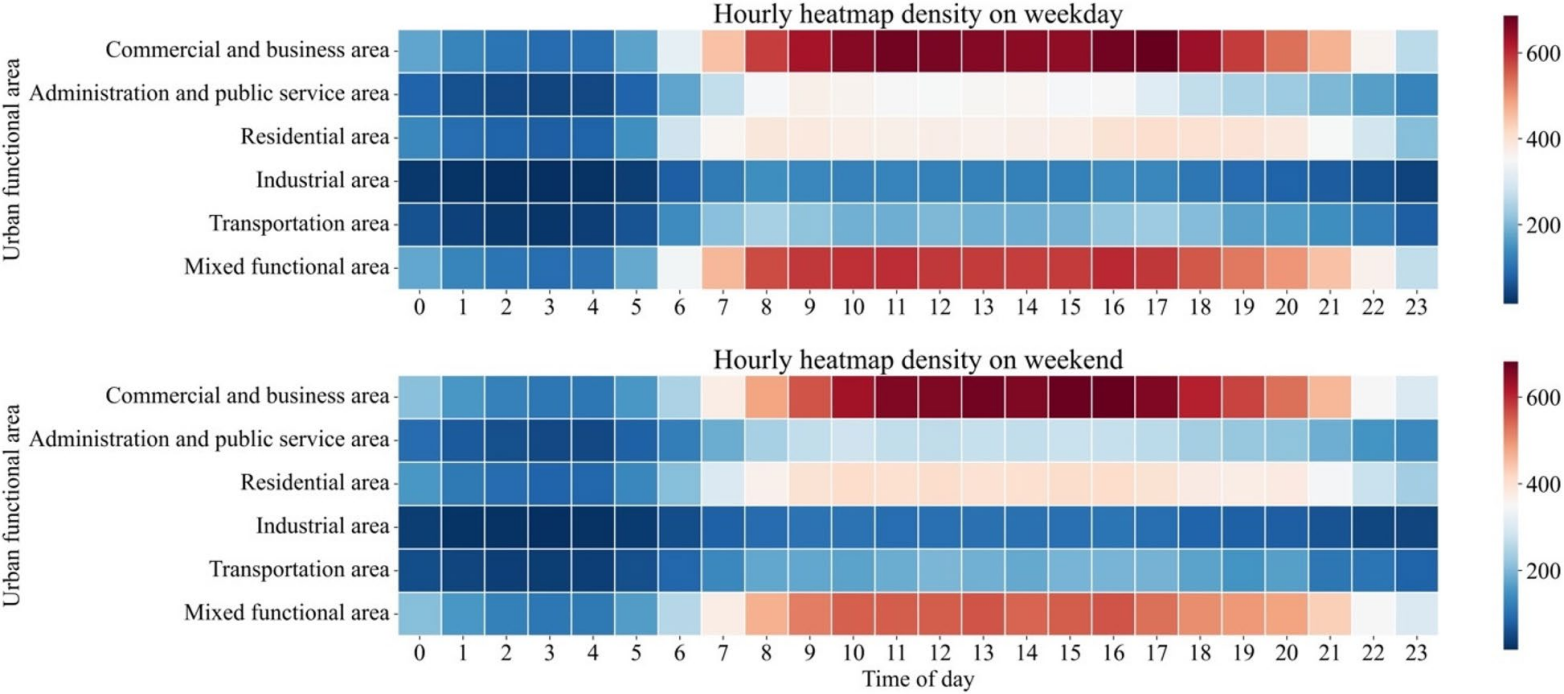
Temporal pattern of the urban vitality on an average weekday and weekend

Figure 6 shows the hourly dynamic pattern of urban vitality on an average weekday and weekend. On the weekday, all urban functional areas exhibited obvious daytime and nighttime differences, with the urban vitality gradually increasing from 6:00 and decreasing after 22:00. Commercial and business areas showed daytime-nighttime variations in urban vitality, with vitality values generally above 500 during the daytime and the highest from 11:00–13:00 and 16:00–18:00. High vitality in commercial and business areas continued until after 22:00. Mixed functional areas exhibited a similar pattern, but their high vitality values were lower than those in commercial and business areas. The hourly urban vitality values in administration and public service areas significantly varied, but the high values were lower, mainly between 8:00 and 16:00. The variation in the vitality value in residential areas was comparable to that in administration and public service areas. The highest values occurred from 7:00–8:00 and 17:00–18:00. Commercial and business areas, mixed functional areas, and residential areas showed higher vitality than other areas at night. The urban vitality was low during both the daytime and nighttime in industrial and transportation areas.

The urban functional areas also revealed pronounced daytime and nighttime differences during the weekend (Fig. 6). Commercial and business areas continued to exhibit the highest variations in urban vitality; however, the high vitality values during the weekend were more notably concentrated in the afternoon compared to those on the weekday. The temporal vitality distribution in mixed functional areas did not differ much from that on the weekday, but the vitality values in these areas were generally lower. The vitality values in administration and public service areas were significantly lower during the daytime than on the weekday. The vitality values in residential areas were higher and peaked around noon. Industrial and transportation areas exhibited similar patterns to those on the weekday.

### Relationship between the built environment and urban vitality

Table 2 summarizes the impact of the built environment on Box-Cox transformed urban vitality variables based on the ordinary least squares (OLS) regression method. The overall goodness of fit values indicated that the functional area type and built environment provided good explanatory power for the transformed urban vitality intensity. In contrast, these factors provided relatively poor explanatory power for the transformed night ratio.

**Table 2:** Regression models of the impact of the built environment on Box-Cox transformed urban vitality

	**Box-Cox transformed Intensity (Tran-I)**		**Box-Cox transformed Variability (Tran-V)**		**Box-Cox transformed Night ratio (Tran-NR)**	
	Coef	Sig	Coef	Sig	Coef	Sig
**POI density**	0.0411	0.000	0.0000	0.719	-0.0001	0.000
**POI diversity**	5.5105	0.000	-0.3000	0.000	0.0157	0.000
**Average building height**	0.4264	0.000	-0.0153	0.000	0.0023	0.000
**Bus stop density**	0.0455	0.000	-0.0008	0.011	0.0001	0.032
**Road network density**	0.0853	0.000	0.0016	0.095	-0.0005	0.000
**Weekend**	-0.6297	0.000	-0.0577	0.000	-0.0014	0.481
**Urban functional area (ref = mixed functional area)**						
**Administration and public service area**	-0.1870	0.570	0.1119	0.000	-0.0180	0.000
**Commercial and business area**	1.0217	0.000	0.0374	0.055	-0.0105	0.001
**Industrial area**	-2.5734	0.000	0.1401	0.000	-0.0242	0.000
**Residential area**	0.7672	0.002	-0.1550	0.000	0.0236	0.000
**Transportation area**	-0.1421	0.752	0.1019	0.006	-0.0248	0.000
**_cons**	4.7926	0.000	-0.5205	0.000	-0.5868	0.000
**Adjusted R**^**2**^	0.5790		0.1590		0.0927	

Dependent variables were box-cox transformed

First, the built environment significantly impacted transformed urban vitality index (Table 2). The higher the POI density was, the higher the Tran-I, which is consistent with the literature (Jacobs, 1961; Zhang et al., 2021), while the Tran-NR slightly decreased. The effects of POI diversity, average building height, and bus station density were similar. The higher the values of these indicators were, the higher the Tran-I, the lower the Tran-V, and the higher the Tran-NR. This indicates that improving the facility diversity, increasing the building height, and enhancing transit accessibility could promote stable socioeconomic activities and nighttime vitality levels (Guo et al., 2021; Ye et al., 2018). A high road network density could help to increase Tran-I but negatively correlated with the Tran-NR, indicating a negative relationship with vitality proportion at night.

The functional area type also determined the urban vitality pattern. After controlling built environment variables, commercial and business areas and residential areas showed higher Tran-I value, and industrial areas were less vibrant than mixed functional areas. All other functional types exhibited a higher Tran-V value than mixed functional areas except for residential areas, which is consistent with the literature indicating that functional use uniformity benefits vitality stability (Guo et al., 2021; Sulis et al., 2018). Only residential areas attained a more significant Tran-NR value than mixed functional areas. All other functional areas obtained a smaller Tran-NR, which confirms the importance of mixed functional areas in increasing nighttime vitality (Zhang et al., 2021; Zheng et al., 2017).

**Fig. 7:**
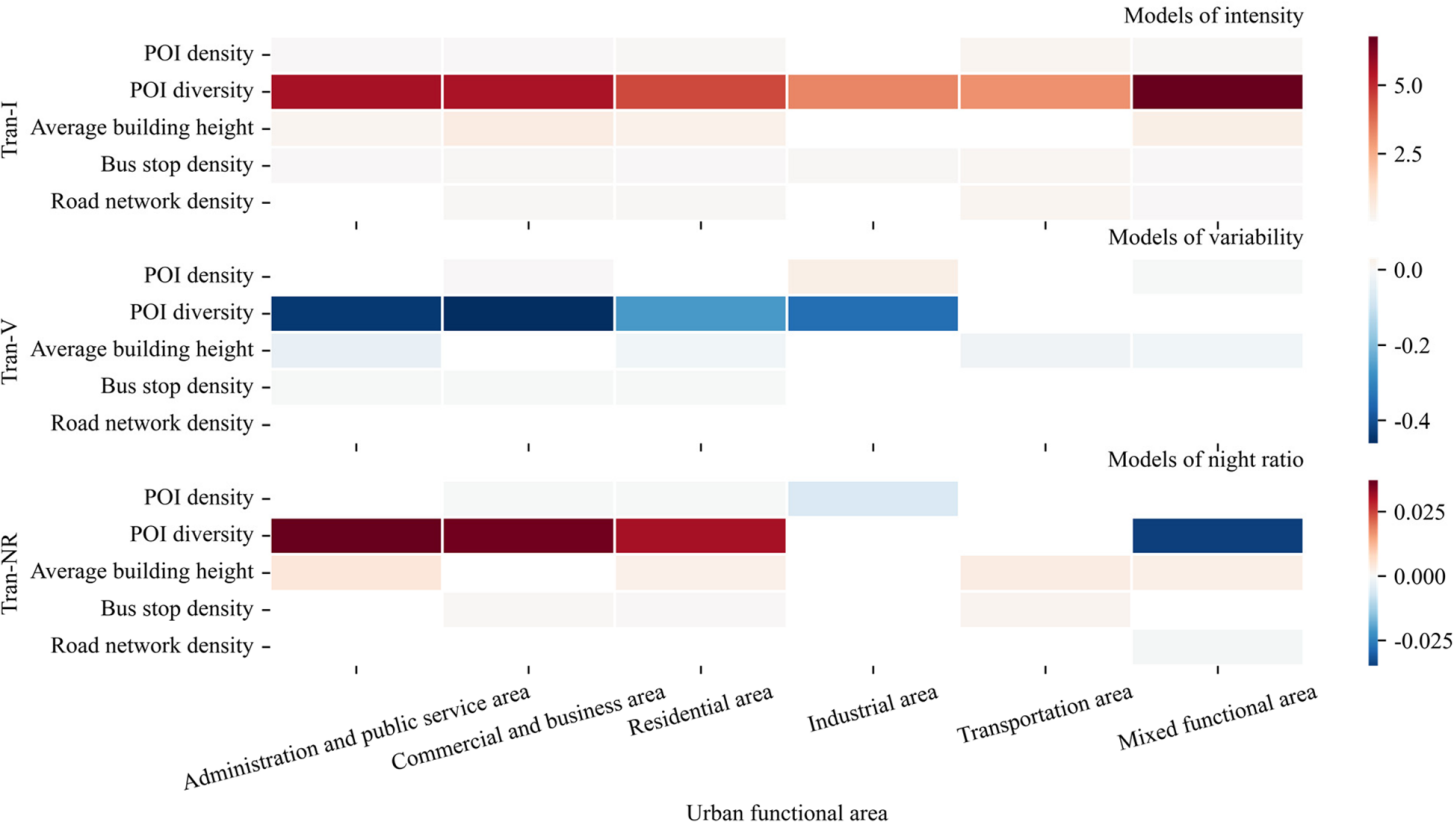
Impact of the built environment on the transformed urban vitality among the various urban functional areas

Figure 7 shows the built environment's impact on transformed urban vitality index among different urban functional areas. Only variables with a significant level above 95% are shown in the figure, with the red color indicating positive effects and the blue color indicating adverse effects. The impact of built environment variables on transformed urban vitality index varied among the different functional areas. Increasing the POI density was beneficial for enhancing the Tran-I in all functional areas except industrial areas. It was positively correlated with Tran-V in industrial areas and commercial and business areas but negatively associated with Tran-V in mixed functional areas. POI density negatively correlated with Tran-NR in commercial and business areas, residential areas, and industrial areas. POI diversity enhancement negatively correlated with Tran-V in all functional areas except for transportation and mixed functional areas. Increasing the POI diversity could facilitate an increase in Tran-NR in administrative and public service areas, residential areas, and commercial and business areas. However, increasing POI diversity was negatively correlated with Tran-NR in mixed functional areas. Raising the average building height enhanced the Tran-I in administration and public service, commercial and business, residential, and mixed functional areas. It negatively correlated with the Tran-V in administration and public service areas and residential areas. The building height increase was positively related to Tran-NR in administration and public service areas, residential areas, transportation areas, and mixed functional areas. The effect of bus stop density on the Tran-I and Tran-V was similar to that of the POI diversity, except for Tran-V in industrial areas. It contributed to an increase in Tran-NR in commercial and business areas, residential areas, and transportation areas. The road network density positively correlated with Tran-I in administration and public service areas, commercial and business areas, transportation areas, and mixed functional areas. But it did not relate to Tran-V in most functional areas. Increasing the road network density negatively impacted Tran-NR in mixed functional areas.

## Conclusions and discussions

Based on Baidu heatmap data, this paper quantitatively measured the dynamic urban vitality in the Central City of Shanghai in terms of intensity, variability, and night ratio. It compared the differences in urban vitality among various urban functional areas and investigated the influence of the built environment on urban vitality. We found that (1) significant differences existed in the intensity, variability, and night ratio of urban vitality among various urban functional areas, reflecting the differences in attracting and maintaining human activities among different urban functional areas. (2) The difference in intensity was more significant than that in the variability and night ratio between an average weekday and weekend. Still, variation differences occurred among various urban functional areas. (3) The built environment significantly affected Box-Cox transformed urban vitality index, but its role varied among different urban functional areas.

In terms of methodology, this paper constructed three dimensions of urban vitality indicators: intensity, variability, and night ratio. It examined the average urban vitality status throughout the day, intraday variation in urban vitality, and diurnal variation in urban vitality. Most studies focus on static indicators of urban vitality, examining the overall characteristics of human activities through the cumulative or average intensity of the urban vitality (Chen et al., 2021; Ye et al., 2018; Yue et al., 2019). In contrast, our study analyzed the ability of urban neighborhoods to attract and sustain human activities at different times using variability and night ratio indexes. We believe these indicators could better enhance our understanding of urban vitality and provide a way to quantitatively evaluate the dynamic characteristics of urban vitality assessment from an integrated perspective.

In practice, we found differences in intensity, variability, and night ratio of urban vitality across urban functional areas. Commercial and business areas and mixed functional areas exhibited higher vitality, moderate variability, and higher nighttime vitality. Administrative and public service areas attained moderate vibrancy but higher variability and lower nighttime vitality values. Residential areas exhibited higher vibrancy levels but the lowest variability and high nighttime vitality values. Industrial and transportation areas demonstrated lower vibrancy, higher variability, and lower nighttime vitality. These findings indicated that the different types of functional areas significantly differed in attracting and maintaining a stable presence of people and activities. However, the urban vitality in the various functional areas varied between an average weekday and weekend. The differences in intensity were more significant than the variability and nighttime vitality. The intensity in administration and public service areas, industrial areas, and mixed functional areas were higher on a weekday. In contrast, the night vitality ratio in residential areas was higher during the weekend.

Our study revealed the relationship between the built environment and Box-Cox transformed urban vitality index among different urban functional areas, which could help support urban planning. As shown in the models, increasing POI mix, building height, and bus stop density were beneficial for increasing transformed urban vitality intensity and nighttime vitality while reducing transformed variability. Increasing POI density and road network density could promote transformed vitality and variability yet reduce the transformed night vitality ratio. However, differences occurred in the impacts of the built environment among different urban functional areas, indicating that the effects of the built environment on different types of human activities varied. Most of the built environment variables positively impacted transformed urban vitality in commercial and business areas, residential areas, and mixed functional areas. However, the built environment's impact on the variation of urban vitality was different. For commercial and business areas and residential areas, the POI diversity and bus stop density were beneficial for enhancing transformed vitality stability and nighttime vitality. In contrast, the POI density had a slightly negative impact. And residential areas with higher building height showed higher transformed vitality stability and nighttime vitality. Taller building height promoted transformed nighttime vitality for mixed functional areas, while higher POI density and diversity could increase transformed vitality variability or reduce transformed nighttime vitality. The POI density, diversity, building height, and transit accessibility positively impacted the transformed vitality intensity for administration and public service areas. And POI diversity and building height positively correlated with transformed vitality stability and nighttime vitality in these regions. The built environment less influenced industrial areas. Therefore, to enhance the urban vitality status in different places, urban planning and design should consider different combinations of built environments, which could meet the needs of various activities at varying times.

There are limitations to this study. First, we used a 500-m grid as the spatial unit, which is limited by our heatmap data. Although a 500-m grid could also reveal spatial patterns, an actual urban block should be used to examine the differences between parcels in the future. Second, we used one-week data to analyze the temporal variation in urban dynamics within a day, which could likely reflect the characteristics of urban dynamics due to the stability of human behavior. However, long-term data may provide more information about the complex temporal urban vitality variations. Future work should consider variation across seasons and years. Third, Due to the impact of the COVID-19 pandemic in early 2020, human activities and social-economic vitality may be lower than before. Fortunately, the focus of this study was the variation of urban vitality over the day and the differences between different urban functional areas, not the absolute level of human activity. Changes in vitality level would not substantially influence our analysis unless such a change displayed an important geographic pattern. Much more work focused on the long-term impact of COVID-19 and temporal variations of urban vitality can be done with heatmap data with a longer time frame. Fourth, we used regression models to analyze the effects of the built environment on the urban vitality in the different urban functional areas and mainly focused on density and mix indicators. Future work should assess the impact of more prosperous indicators, especially urban design elements.

## Data Availability

The datasets generated during and analyzed during the current study are available from the corresponding author on reasonable request.
